# Case Report: Familial Pseudohyperkalemia Due to Red Blood Cell Membrane Leak in a Chinese Patient

**DOI:** 10.3389/fmed.2022.825174

**Published:** 2022-03-17

**Authors:** Weijue Xiong, Junxian Song, Zhihong Yue, Lin Pei, Yang Liu, Jiangtian Chen, Hong Chen

**Affiliations:** ^1^Department of Cardiology, Peking University People's Hospital, Beijing Key Laboratory of Early Prediction and Intervention of Acute Myocardial Infarction, Center for Cardiovascular Translational Research, Beijing, China; ^2^Department of Clinical Laboratory, Peking University People's Hospital, Beijing, China; ^3^Department of Hematology, Peking University People's Hospital, Beijing, China

**Keywords:** RBC membrane disease, temperature, potassium leak, familial pseudohyperkalemia, *ABCB6* gene

## Abstract

Hyperkalemia is a critical condition requiring careful evaluation and timely intervention. Many conditions could manifest as pseudohyperkalemia and it's important to differentiate them as inappropriate potassium-lowering therapy might lead to detrimental outcomes. A 56-year-old female was admitted for hyperkalemia (5.62–8.55 mmol/L). She had no symptoms or signs of hyperkalemia. A comprehensive work-up of hyperkalemia retrieved no valuable findings. Her blood samples underwent incubation tests at different temperatures and revealed temperature-dependent potassium leaks from red blood cells. Based on all test results, a diagnosis of hyperkalemia caused by red blood cell membrane defects was suspected. Whole-genome sequencing revealed a heterozygous c.1123C>T (p. R375W) mutation in the *ABCB6* gene and confirmed the diagnosis of familial pseudohyperkalemia (FP). FP is an inherited benign condition in which red blood cells have increased cold-induced permeability to potassium. The patient was discharged with no additional treatment and she was suggested avoiding blood donation.

## Introduction

Hyperkalemia, defined as plasma potassium (K^+^) concentration exceeding 5.5 mmol/L (1), occurs in 1.1% to 10% of inpatient admissions (2–4). Severe hyperkalemia (K^+^≥6.5 mmol/L) is significantly correlated with increased mortality risks (5). The plasma potassium concentration is determined by potassium intake, the distribution of potassium between intracellular space and extracellular fluids, and urinary potassium excretion. Pseudohyperkalemia should be considered when patient history, physical examination and laboratory tests do not explain the level of hyperkalemia.

Here, we report the case of a 56-year-old female patient admitted for hyperkalemia as high as 5.62–6.79 mmol/L. On investigation, she had no symptoms or signs of hyperkalemia, and multiple repeated tests confirmed a highest level of 8.55 mmol/L. These repeated blood samples were collected in serum separator gel tubes, stored at room temperature (about 20°C), delivered manually and measured within 2 h. A comprehensive work-up of hyperkalemia was conducted; patient's blood samples underwent incubation tests at different temperatures compared with samples from acute myeloid leukemia (AML) patients and healthy volunteers. We carefully analyzed the process of venepuncture, specimen delivery and biochemical analyzer testing and suspected an uncommon kind of pseudohyperkalemia. A definite diagnosis of familial pseudohyperkalemia (FP) was confirmed by whole-genome sequencing, and a heterozygous c.1123C>T (p. R375W) mutation in *ABCB6* gene was identified.

## Case Presentation

This study adhered to the tenets of the Declaration of Helsinki (6). The patient provided informed consent for the publication of this case report and associated images.

A 56-year-old Chinese female was admitted for intermittent hyperkalemia (5.62–6.79 mmol/L, normal range 3.5–5.5 mmol/L, 12 times out of 26 measurements) with no other electrolyte abnormality on routine check-ups. She denied palpitation, chest discomfort, or muscle weakness. Her dieting habits and urine volume were normal, and she denied a medication history of potassium supplementation or angiotensin-converting enzyme inhibitors/or angiotensin receptor blockers. The patient went to our emergency department, underwent immediate electrocardiograph and showed no cardiac conduction abnormalities or arrhythmias. She was given sodium polystyrene sulfonate (a gastrointestinal cation exchanger) to reduce serum potassium to within 5.5 mmol/L. She reported a weight loss of 20 kg from dieting during the past 6 months. She had mild coronary atherosclerosis, and was on aspirin, atorvastatin and isosorbide mononitrate. She received cesarean surgery in 1988 and had a previous smoking history. Her family history included hypertension, diabetes and coronary heart disease. Her vital signs were stable, her heart rate was 70 beats per minute and her body mass index was 22.0 kg/m^2^. Other physical examination was unremarkable.

A series of tests were performed to investigate the causes of hyperkalemia. Her white cell count was 5,910/μL (normal range 3,500–9,500/μL), hemoglobin was 13.2 g/dL (normal range 11.5–15 g/dL) and platelet count was 197,000/μL (normal range 125,000–350,000/μL) by measurement of EDTA anticoagulation whole blood. Her serum electrolyte profiles (including sodium, chlorine, calcium and magnesium), liver and renal function were assessed following standard methods on an AU5832 automated analyzer (Beckman Coulter Inc., Brea CA, USA) and the results were within normal limits. Her estimated glomerular filtration rates, 24 h urine potassium output and adrenal function tests were normal. Her glycosylated hemoglobin was 5.4% (normal range 4.0–6.0%) and oral glucose tolerance tests showed normal glucose tolerance and insulin secretion patterns. Her blood smear showed normal erythrocyte morphology. The mean corpuscular volume (MCV) was 95.3 fL (82–100 fL), reticulocytes were 68,600/μL and 1.68% by proportion, and erythropoietin was 21.8 mIU/mL (normal range 5.4–31 mIU/mL). Her hemoglobin electrophoresis result was normal. Meanwhile, we tested plasma lactate dehydrogenase (LDH), haptoglobin and free hemoglobin and ruled out hemolysis. The potassium level in immediate blood gas analysis was 4.3 mmol/L. In all, a diagnosis of pseudohyperkalaemia was highly suspected.

**Figure 1:**
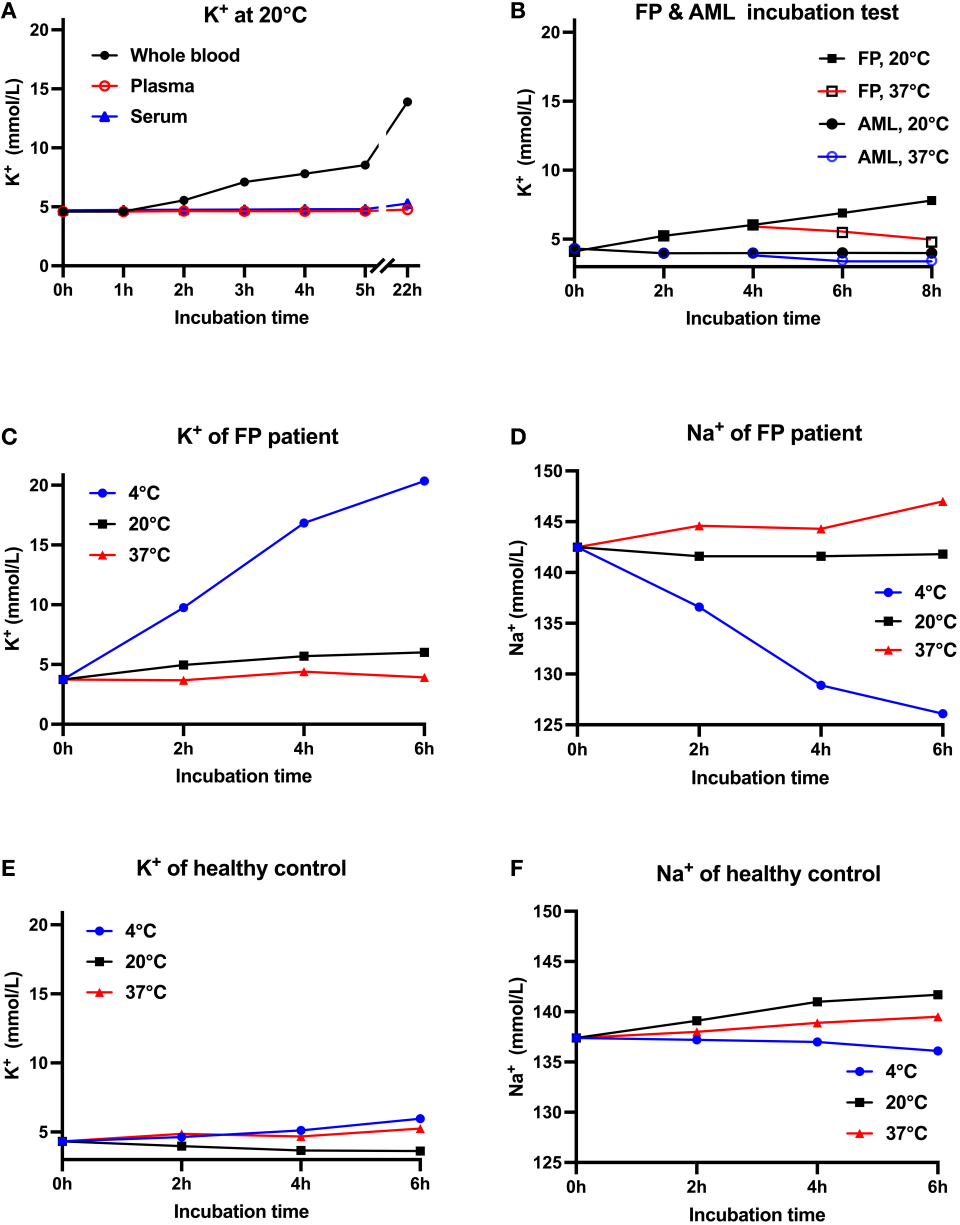
Relationship between K^+^ concentration, blood sample types and storage temperatures. **(A)** Reassessment of the patient's potassium levels at 1, 2, 3, 4, 5, and 22 h after blood collection. Baseline K^+^ concentration in whole blood, plasma and serum samples was normal. Whole blood K^+^ increased with storage time, while isolated plasma and serum K^+^ showed minor changes at 20°C. **(B)** Two whole blood samples from the patient and two samples from AML patients were first stored at 20°C for 4 h and then respectively stored at 20°C or 37°C. This patient's blood K^+^ gradually increased at 20°C but decreased at 37°C, while AML patients' K^+^ basically remained unchanged **(C–F)**. K^+^ & Na^+^ concentration from the FP patient's **(C,D)** and healthy control's **(E,F)** blood samples stored at 4, 20, and 37°C. Compared with healthy controls, the FP patient's measured K^+^ increased dramatically at 4°C, while potassium at 20 and 37°C merely showed slight fluctuations. All tests were conducted once. AML, acute myeloid leukemia.

We arranged several tests to elucidate the relationship between potassium level and blood sample types as well as temperature (Figure 1). Venous whole blood and plasma samples were drawn in tubes containing heparin sodium as an anticoagulant, while serum samples were collected in gel separator tubes. All blood samples were transported manually by standard practice and stored at prearranged time interval and temperature. Plasma and serum samples were isolated after centrifugation at 3,000rpm for 5 min. Potassium and sodium were measured simultaneously by the ion-selective electrode method on an AU5832 biochemical analyzer. All tests were conducted only once considering the large amount of blood needed. Firstly, we noticed that this patient's whole blood potassium concentration increased gradually with storage time and peaked at 13.89 mmol/L after standing overnight at room temperature (RT, ~20 degrees centigrade). In contrast, isolated blood plasma and serum potassium samples showed minor changes at room temperature for up to 22 h. This indicated that spiked potassium was released from blood cells (Figure 1A). In test B, blood samples from AML patients were experimentally used as control because large amounts of vulnerable white cells from AML patients were more likely to influence potassium after extended storage time (7, 8). All whole blood, samples were put to 37°C incubation after 4 h at RT. The patient's potassium level reduced from 6.03 mmol/L to 4.79 mmol/L after warming-up, whereas samples from AML patients did not show such reversibility (Figure 1B). Later, the patient's (Figures 1C,D) and a healthy volunteer's (Figures 1E,F) blood samples were collected and stored separately at 4, 20, and 37 °C, while blood potassium levels were tested after 2, 4 and 6 h. The concentration of potassium in the blood samples from the patient increased dramatically from 3.75 mmol/L to 20.35 mmol/L at 4°C, with sodium level reduced from 142.0 mmol/L to 126.0 mmol/L; while the potassium in the volunteer's samples increased from 4.32 mmol/L to 5.97 mmol/L and sodium decreased from 137.4 mmol/L to 136.1 mmol/L. Potassium in blood samples stored at 20 and 37°C merely showed slight fluctuations. Therefore, we highly suspected this patient to have RBC membrane defect conditions. The degree of hemolysis over time for the blood samples was evaluated by simultaneous LDH levels, which were within normal limits in all tests (LDH data are not shown). The low temperature might reversibly increase this patient's RBC membrane permeability and lead to more potassium leak.

Whole-genome sequencing was conducted and revealed a heterozygous missense mutation in her *ABCB6* gene that caused a malformation variant in the transmembrane *ABCB6* protein (c.1123C>T, p.R375W). The *ABCB6* gene is responsible for an autosomal dominant inheritary condition, familial pseudohyperkalemia. The variant was previously described and functionally validated as causative of FP (9, 10). The patient's parents passed away and her only daughter was unavailable for genetic analysis. In the end, the patient was discharged with no particular medication, and she was suggested to avoid blood donation because of her altered RBC cation permeability.

## Discussion

This is the first ascertained FP case in China. FP is a hereditary benign condition that calls for clinical recognition. Erroneous treatment of pseudohyperkalaemia, as was the case in this patient, may precipitate hypokalemia, which may present with muscle weakness, myalgia, tremor, ascending paralysis, intestinal paralysis, respiratory failure, cardiac failure and arrhythmias (11). Luckily in our case, we stopped sodium polystyrene sulfonate soon enough to avoid such consequences.

An understanding of potassium physiology is helpful when approaching patients with hyperkalemia. Total body potassium stores are approximately 3,000 mmol or more and 50 to 75 mmol/kg body weight (12). The Na^+^-K^+^-ATPase pump maintains the distribution of potassium in intracellular and extracellular fluids across the cell membrane, by pumping sodium out of and potassium into cells. Therefore, the plasma potassium concentration is determined by between potassium intake, the distribution of potassium between cells and extracellular fluids, and potassium excretion. There are two major non-iatrogenic mechanisms of hyperkalemia: increased potassium release from cells (e.g., leukocytosis and thrombocytosis, severe hyperglycemia, rhabdomyolysis) and reduced potassium excretion in urine (e.g., hypoaldosteronism, acute or chronic renal failure).

Pseudohyperkalemia should be suspected when there are no aforementioned causes of hyperkalemia in patients with no related clinical or electrocardiographic manifestations. It refers to elevated serum potassium due to potassium movement out of the cells during or after the blood specimen is drawn (13). Serum potassium usually exceeds plasma potassium because of cell lysis and potassium release in the clotting process. Singh et al. suggested that the serum potassium exceeds the plasma potassium by more than 0.4 mmol/L for pseudohyperkalaemia to be considered (14). One clue to the possible presence of pseudohyperkalemia is wide variability in repeated measurements of the serum potassium concentration (e.g., from 5 to 6.5 mmol/L, often including some normal values). As concluded in Table 1 [adapted from Meng et al. (15)], patient factors, inappropriate blood sample drawing, transportation and storage, and sample measuring processes could bring about falsely elevated plasma potassium. In patients presenting with elevated potassium levels, it is helpful to compare potassium values from separated plasma/serum vs. whole blood samples after extended storage time as shown in our case. While separation of serum takes relatively more time, immediate electrolyte analysis from arterial blood gas could serve as a good alternative to avoid electrolytes release from blood cells.

**Table 1:** Common causes of pseudohyperkalaemia.

**1. Patient factors**
**•Leukocytosis, thrombocytosis** **•RBC membrane leaking disease, e.g., familial pseudohyperkalaemia, hereditary spherocytosis**
**2. Blood sample drawing**
**•Contamination from potassium-containing (e.g., K_2_-EDTA) collection tubes**
**•Prolonged tourniquet application before venepuncture**
**•Repeated fist clenching and vigorous shaking of blood tubes**
**•Drawing above intravenous site**
**3. Blood sample transportation and storage**
**•Cooling of the sample**
**•Prolonged length of storage**
**•Transport through pneumatic tube system**
**4. Sample measurement**
**•Recentrifugation**

FP is characterized by increased permeability to potassium through the red cell membrane at cold temperatures associated with essentially normal hematology, no significant hemolysis (9). Affected patients demonstrate normal RBC membrane permeability at body temperature (37°C) but FP lead to measured potassium elevation after potassium leaks out of RBCs in blood stored below 37°C (9). Some patients' RBCs also show macrocytosis because of cell swelling after 24 h on ice (16). Potassium flux could be measured using ^86^RbCl as a tracer in the presence of ouabain and bumetanide, as they inhibit the Na^+^-K^+^ ATPase and Na^+^-K^+^-2Cl^−^ cotransporter (17). The activity of the Na^+^, K^+^-cotransport system in pseudohyperkalaemics was more temperature sensitive than that of normal controls and affected individuals showed a greater passive permeability to potassium at low temperatures (16). In addition to FP, a variety of leaky RBC disorders have been discovered, including dehydrated hereditary stomatocytosis, overhydrated hereditary stomatocytosis, and cryohydrocytosis (18). These conditions differ from FP in that they are symptomatic; they often present with altered RBC morphology and hemolytic anemia and are sometimes associated with iron-overload, neurological symptoms, neonatal ascites and/or thrombosis complications after splenectomy. Besides, by measurements of net potassium movements, using ^86^Rb influx with ouabain and bumetanide, several temperature-dependent patterns of cation leak have been characterized and could be used to differentiate the disease spectrum (19). FP stands out among the leaky RBC disorders for its mild clinical and hematological phenotype and its minimal changes in cell shape. The temperature dependences of the cation leaks in the FP pedigrees include U-shaped, shallow slope and shoulder types (20), which means different leak rates at different temperatures. Interestingly, our incubation test (Figure 1B) showed some reversibility of potassium leaking, consistent with observations by Bawazir et al. (21). However, warming the FP RBCs did not reduce potassium back to normal levels. An explanation for the partial reversibility of the potassium leak is that, as the leak is a bi-directional diffusional process, rewarmed RBCs might leak less potassium and reabsorb some potassium back into cells. Determination of the full extent of the reversibility of the leak will need more experimental evidence.

The molecular bases of a number of FP pedigrees have been determined recently. FP is an autosomal dominant conditioncaused by mutations in the ATP-binding cassette, subfamily B, member 6 gene (*ABCB6*) on chromosomes 2q35-36 and 16q23-qter (9). *ABCB6* encodes erythrocyte membrane protein and bears Langereis (Lan) blood group antigen and was shown to play a role in exporting porphyrin from red blood cells. *ABCB6* is also expressed as a mitochondrial membrane protein and has participated in porphyrin transport and heme biosynthesis. There are several reported missense mutations for *ABCB6* in FP, namely, heterozygous R375Q (c.1124 G>T) of Flemish origin (FP Lille), R375W (c.1123C>T) of Pakistani [FP Falkirk, remarkable for increased MCV (9, 10)] and Bangladeshi (FP East London) origin, R723Q (c.2168G>A) of Welsh origin (FP Cardiff) (21), and R276W (c.826G>T) variant from Irish population (10) as previously described. Expression of *ABCB6* mutants showed no changes in RNA or polypeptide levels but somehow changed the membrane permeability to potassium. Our case is the first reported FP case in the Chinese population, and it coincides with FP Falkirk genotype-a heterozygous c.1123C>T (p. R375W) mutation in *ABCB6*. However, unlike FP Falkirk (16), our patient showed a normal MCV. Unfortunately, we were not able to conduct a thorough survey of her pedigree; The heterozygous R723Q SNP (rs148211042) was reported as present in approximately 1: 500 UK donors and the R375W SNP (rs764893806) is thought to be present in approximately 1:35,000 UK donors; the variant R276W was present in 0.3% (1/327) of blood donors from South Italy (21). The estimated accumulative frequencies of FP individuals are 1/785 in Asian population (22). Since few FP cases have been reported in Asia, FP prevalence might be substantially understated. This calls for disease recognition and thorough investigation, as it might be common and could bring about improper potassium-lowering treatment. Transfusion-associated hyperkalemic cardiac arrest is a serious complication of rapid RBC administration (23). If a blood donor has FP, the RBC concentrates in refrigerated storage are likely to release a large amount of potassium that could be fatal for neonates/infants or adults with large transfusion. Thus, awareness should be raised about recognition of FP patient donations and prevention of transfusion-associated hyperkalemia (21).

## Conclusion

Pseudohyperkalemia refers to potassium leaks from RBCs during or after the blood specimen is drawn. It is common in clinical settings and should be considered when patients show unmatched symptoms or fluctuating laboratory results. Specifically, it might indicate critical hematological conditions such as leukocytosis, thrombocytosis and RBC membrane leaking disease. FP is a benign condition and is often diagnosed with fluctuating high potassium as the first symptom. Inappropriate potassium-lowering therapy might lead to detrimental outcomes. In addition, asymptomatic individuals with FP may become potential blood donors, which could be detrimental for neonates and infants receiving their donations. More experiments should be conducted to find out whether incubation of RBCs at 37°C for some time before blood transfusion could drive extracellular potassium back to RBCs.

## Data Availability Statement

The datasets presented in this article are not readily available because of ethical/privacy restrictions. Requests to access the datasets should be directed to the corresponding author.

## Ethics Statement

Ethical review and approval was not required for the study on human participants in accordance with the local legislation and institutional requirements. The patient/participant provided her written informed consent to participate in this study.

## Author Contributions

WX and JS analyzed and interpreted the clinical data. ZY, LP, and YL designed the data analysis and collected the data. JS, JC, and HC revised the manuscript. All authors have read and approved the final manuscript.

## Funding

This research received funds from Capital's Funds for Health Improvement and Research project 2020-2-4084.

## Conflict of Interest

The authors declare that the research was conducted in the absence of any commercial or financial relationships that could be construed as a potential conflict of interest.

## Publisher's Note

All claims expressed in this article are solely those of the authors and do not necessarily represent those of their affiliated organizations, or those of the publisher, the editors and the reviewers. Any product that may be evaluated in this article, or claim that may be made by its manufacturer, is not guaranteed or endorsed by the publisher.
